# Supplementary material for: Charge-noise spectroscopy of Si/SiGe quantum dots via dynamically-decoupled exchange oscillations
Source: Nat Commun. 2022 Feb 17;13:940. doi: 10.1038/s41467-022-28519-x (PMC8854405; doi:10.1038/s41467-022-28519-x)
Supplement: Supplementary file 1 — Supplementary Information [file 41467_2022_28519_MOESM1_ESM.pdf]

Supplementary Information:
Charge-noise spectroscopy of Si/SiGe quantum dots via dynamically-decoupled exchange oscillations

Elliot J. Connors,[1] JJ Nelson,[1] Lisa F. Edge,[2] and John M. Nichol[1,*]

[1] *Department of Physics and Astronomy, University of Rochester, Rochester, NY 14627*
[2] *HRL Laboratories LLC, 3011 Malibu Canyon Road, Malibu, California 90265, USA*

[*] john.nichol@rochester.edu

α_{P1} (eV/V)	0.098
α_{P2} (eV/V)	0.112
α_{S} (eV/V)	0.055

Supplementary Table 1: Relevant lever arms of the S-T_0 qubit device. α_{i} is the lever arm corresponding to gate i.

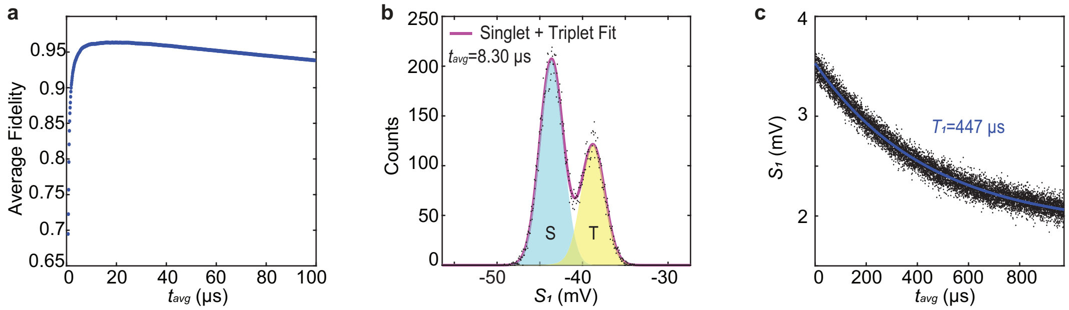

Supplementary Figure 1: **Readout characterization.** **a** Average singlet-triplet readout fidelity as a function of integration time t_{avg}. **b** Histogram of 10,000 single-shot measurements of randomly-prepared joint-spin states analyzed with an integration time $t_{avg} = 8.3$ μs and having an average fidelity of 96%. Blue and yellow peaks correspond to singlet and triplet states, respectively. **c** Difference of the average measured signal after singlet- and random-initialized states as a function of integration time. We observe $T_1 = 447$ μs.

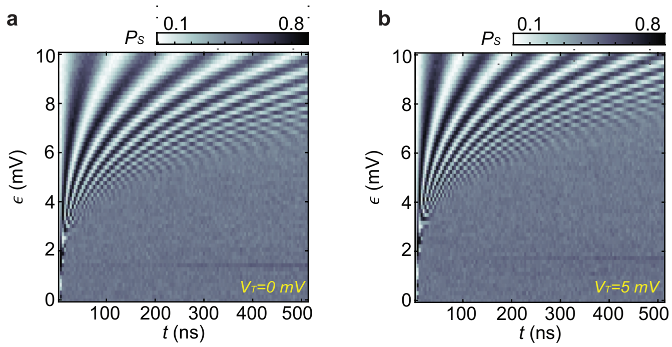

Supplementary Figure 2: **FID measurement to confirm $|dJ/d\epsilon| > |dJ/dV_T|$.** **a** Exchange oscillations versus ϵ with $V_T = 0$ mV. **b** Exchange oscillations versus ϵ with $V_T = 5$ mV. The data in **a** and **b** were acquired on the same device tuned to the same charge transition as in the main text, but in a different dilution refrigerator. By comparing the data in **a** and **b**, we confirm that $|dJ/d\epsilon|$ is at least five times larger than $|dJ/dV_T|$ at relevant values of ϵ.

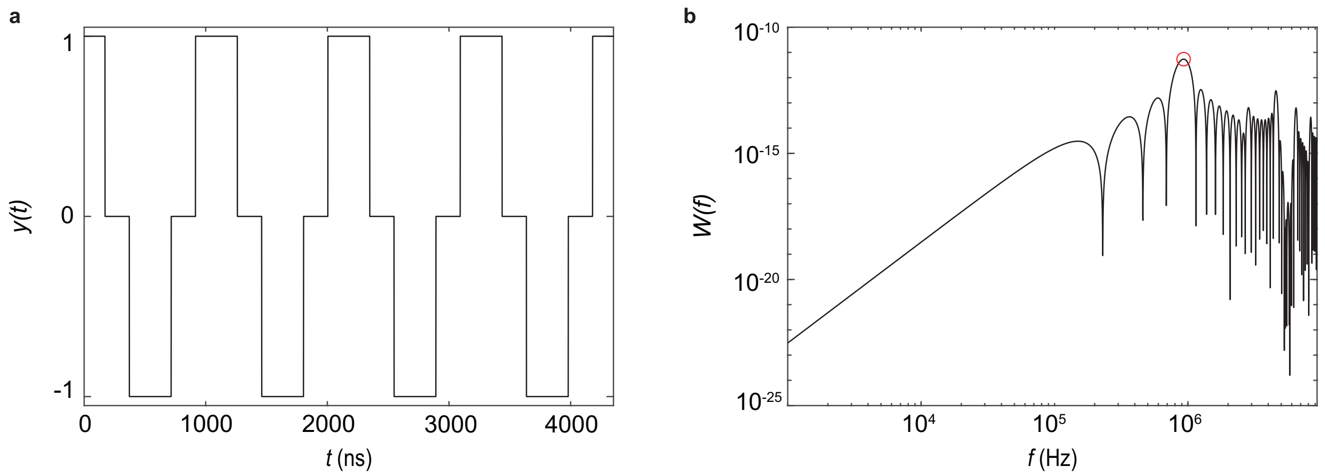

Supplementary Figure 3: **CPMG filter function. a** $y(t)$ corresponding to a CPMG experiment with $n_\pi = 8$, $t_\pi = 200$ ns, and $\tau = 2752$ ns. **b** Spectral weighting function, $W(f)$, corresponding to $y(t)$ in **a**. A red circle is plotted at the maximum value of $W(f)$, corresponding to the frequency at which the experiment is most sensitive to noise.

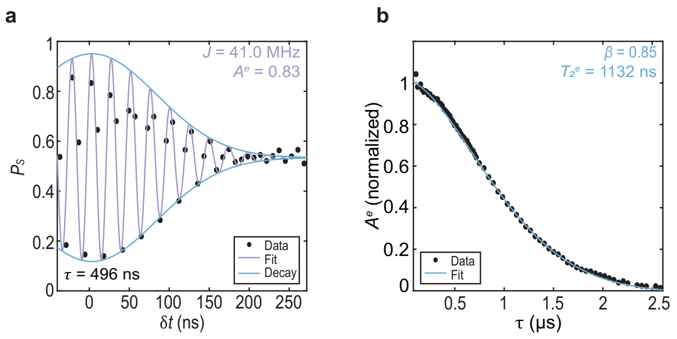

Supplementary Figure 4: **Spin-echo data analysis.** **a** Representative example of an echo signal as a function of δt. J and A^e are extracted from a fit of the data (purple). **b** Echo amplitude decay. The decay are well described by a function of the form $\exp[-\chi(\tau)]$ suggesting decoherence resulting from $S = A/f^\beta$ noise. Because the deviations in β are most pronounced at the beginning and the end of the decay curves [1], we increase the measured point density for small τ resulting in a more reliable fit.

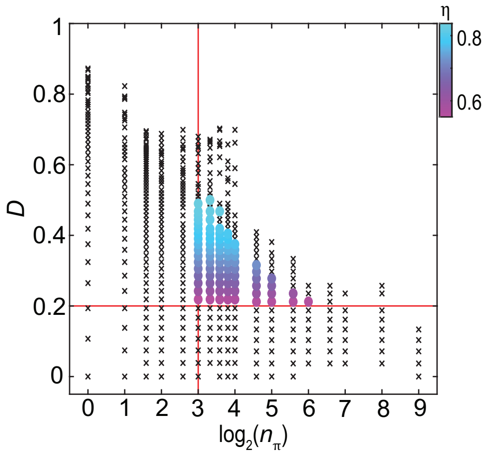

Supplementary Figure 5: **Estimation of the error in the extraction of noise from CPMG measurements.** Plot of the D and $\log_2(n_\pi)$ values associated with each time step in each CPMG experiment. Black x's correspond to data satisfying at least one of: $D < 0.2$ (red horizontal line), $n_\pi < 8$ (red vertical line), $A^{n_\pi} < 0.15$, or $A^{n_\pi} > 0.85$. We do not extract information about the noise from these points. Colored circles correspond to data from which we calculate the corresponding noise spectrum. They are colored according to $\eta = S_{est}(f_0)/S(f_0)$.

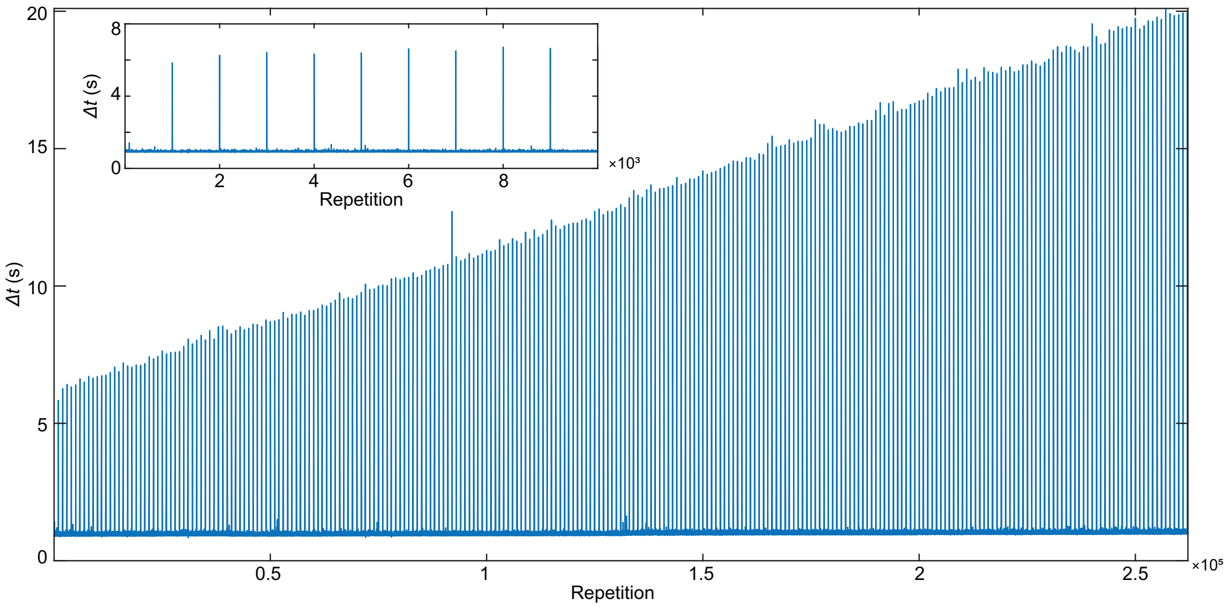

Supplementary Figure 6: **Sampling rate in three-day FID experiment.** Plot of Δt, the time between exchange-oscillation repetitions, as a function of the repetition number in the three-day FID experiment. The inset shows just the first 10,000 repetitions of the same data. For nearly all repetitions, $\Delta t \approx 0.97$ seconds, but once every 1,000 repetitions, the total acquired data is saved to a file causing a delay between repetitions. As the total amount of data increases, it takes longer to write the data to the file, and we see the delay time increase.

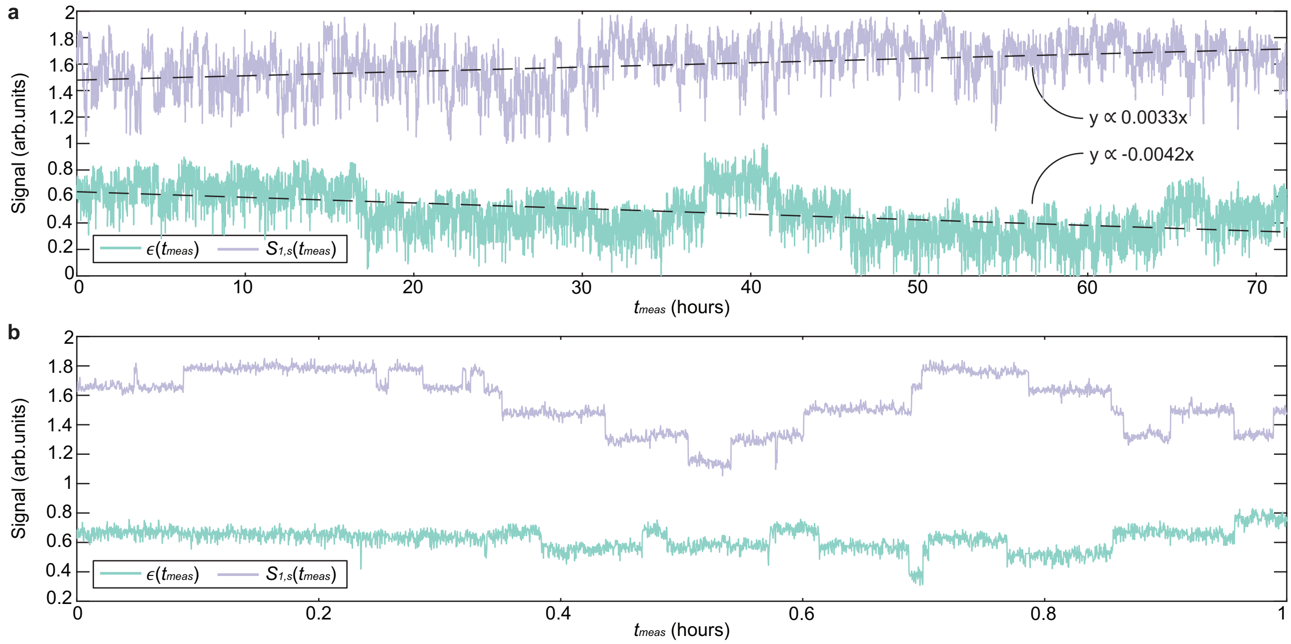

Supplementary Figure 7: **Temporal charge-noise correlations.** **a** Normalized signals of the detuning noise of the qubit (green) and chemical potential noise of the charge sensor (purple). The dashed lines are straight line fits to the data. **b** The same data shown in **a** plotted for only the first hour of the experiment. In both **a** and **b**, $S_{1,s}(t_{meas})$ is offset for clarity.

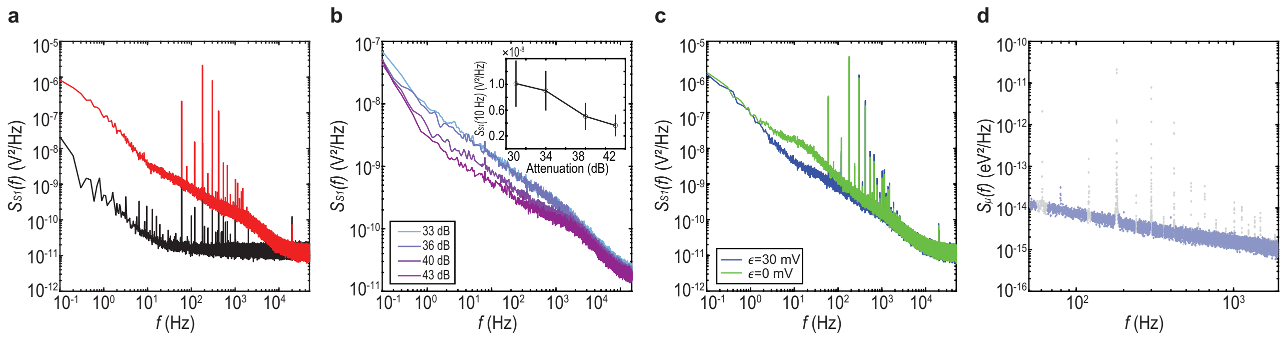

Supplementary Figure 8: **Charge-sensor spectra.** **a** Spectra acquired when $|dS_1/dV_S|$ is large (red), and when $|dS_1/dV_S| \approx 0$ (black) illustrating the background noise in the measurement setup. **b** Spectra acquired at $\epsilon = 30$ mV with differing amounts of room-temperature attenuation applied to the rf carrier. Spectra are down-sampled and smoothed via a five-point moving average. Inset shows the magnitude of the noise averaged between 5 and 15 Hz, where differences in the spectra are most pronounced, as a function of applied attenuation. Error bars represent the standard deviation in the data from 5 to 15 Hz. We observe negligible changes above 43 dB of attenuation used. **c** Spectra acquired with $\epsilon = 30$ mV (blue) and $\epsilon = 0$ mV (green) illustrating tuning dependence of measured noise. **d** Plot of the charge-sensor spectrum corresponding to the data shown in Figure 5. Data shown in Figure 5 are reproduced in purple, while the data near multiples of 60 Hz, which were omitted in the main text, are shown here in grey.

SUPPLEMENTARY NOTE 1: HADAMARD GATE CALIBRATION

The Hadamard gate for an S-T_0 qubit represents an evolution under a Hamiltonian where $J = \Delta B_z$. We calibrate our Hadamard gate using a three-step procedure. First, we extract the approximate magnitude of ΔB_Z via a FID measurement at large ϵ such that $\Delta B_Z > J$. Next, with a fixed value of V_T, we measure the qubit oscillation frequency versus ϵ in the range of ϵ where $J(\epsilon, V_T) \approx \Delta B_z$ (chosen based on the approximate ΔB_Z magnitude and knowledge of the $J(\epsilon, V_T)$ landscape from previous FID measurements), and determine times, $t_\pi(\epsilon)$, corresponding to π rotations at each ϵ. Then, for each different value of ϵ, we apply this π rotation to an initialized $|S\rangle$. We allow the qubit to evolve under J for a variable time, and then apply the same π rotation discussed above before readout. The visibility of the exchange oscillations is a maximum when the prepared state lies in the $x - y$ plane. We therefore choose the detuning and time of the Hadamard gate as the ϵ value and corresponding π rotation time which give the largest-visibility exchange oscillations. Importantly, this calibration procedure relies on optimizing the visibility of rotations about the z-axis before and after applying H. We empirically find that this procedure also suffices to tune the composite $X = HZH$, along with a separate calibration of the Z gate. Typical H gate durations are 88-96 ns. Neglecting effects of pulse rise times, these times suggest that $\Delta B_z \approx 3.85$ MHz.

In practice, we recalibrate the Hadamard gate a short time prior to all echo and CPMG measurements because the refocusing pulses are highly sensitive to how well the gate is calibrated. However, recalibration once per day is usually sufficient for state preparation and readout purposes. Hyperfine fluctuations associated with residual ^{29}Si will have a negative impact on the H gate, and we expect better performance in isotopically-purified Si.

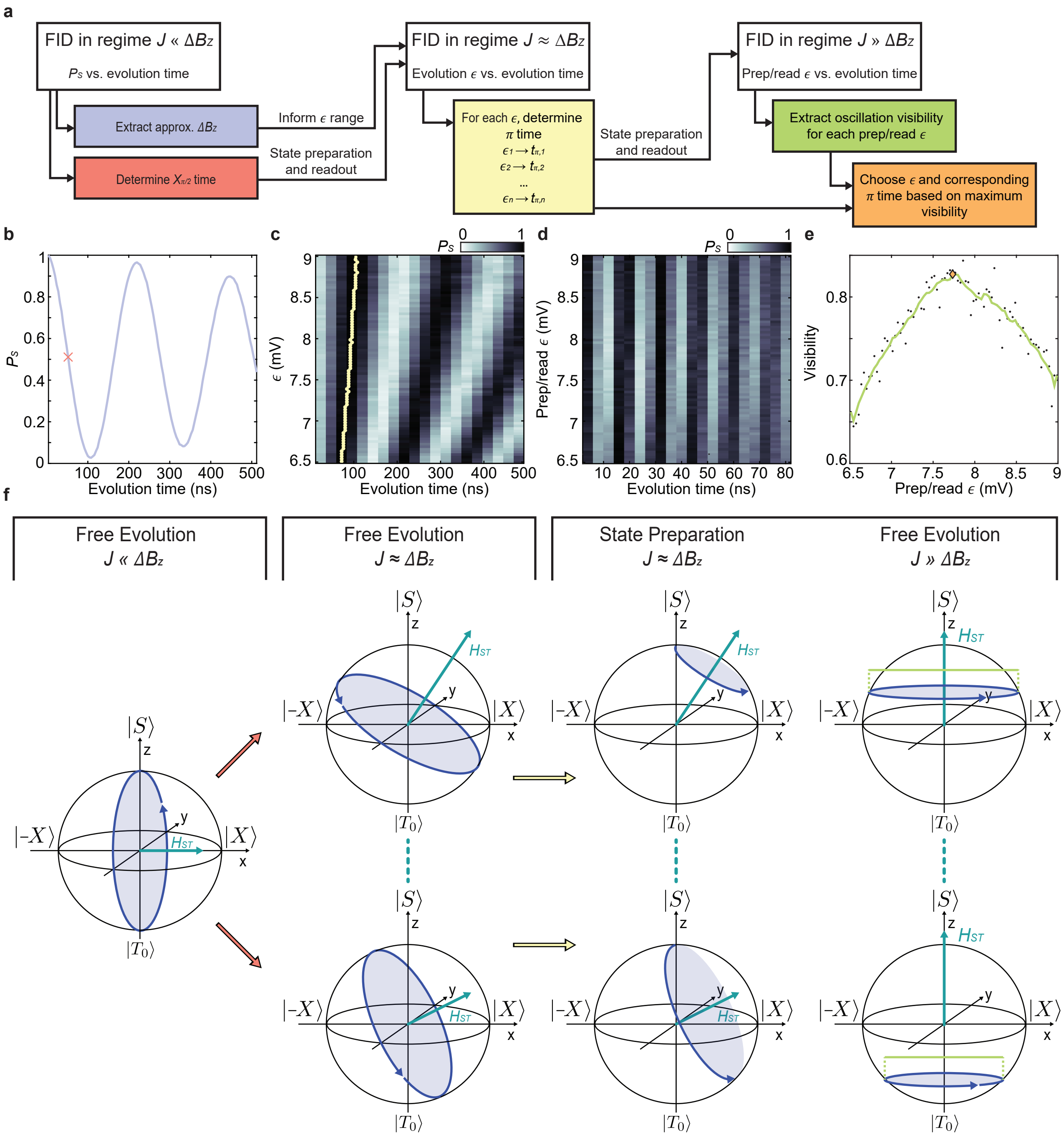

Supplementary Figure 9: **Automated Hadamard gate calibration procedure.** **a** Overview of the measurements and analysis involved in calibrating the Hadamard gate. The three measurements are shown in white boxes. Analysis of the respective measurements are shown in colored boxes below. **b** FID oscillations measured at large ϵ. From these, we extract the approximate magnitude of ΔB_Z, and determine the time required to perform an approximate $X_{\pi/2}$ gate (red x). **c** FID oscillations in regime where $J \approx \Delta B_Z$. The veritcal axis is the value of ϵ at which the free evolution occurs. For this measurement, the initial state is prepared and read out by applying the $X_{\pi/2}$ immediately before and after the free evolution of an initialized $|S\rangle$, which ensures that we observe FID oscillations at ϵ values where J is both larger and smaller than ΔB_Z. From these measurements, we extract the qubit oscillation frequency for each ϵ and determine associated π times (yellow dots) to within the timing resolution of the AWG (4 ns). **d** FID oscillations at large, fixed $J(\epsilon, V_T)$. The veritcal axis is the value of ϵ at which we apply the π pulse immediately prior to and after the free evolution in order to prepare and read out the qubit state. **e** Plot of the visibility of FID oscillations shown in **d** as a function of the preparation and readout ϵ. We smooth the data via a moving average (green line). The ϵ value corresponding to the maximum of the smoothed visibility is identified as the optimal ϵ value for the Hadamard gate. **f** Bloch sphere schematic of the three calibration measurements outlined in **a**-**e** above. The first and second columns from left correspond to the free evolution portion of the first and second measurements, respectively (state preparation and readout not shown). The third and fourth columns from left show the state preparation and free evolution segments of the third measurement. The visibility corresponds to the width of the oscillation plane in the fourth column from left (green bar).

SUPPLEMENTARY NOTE 2: t_{meas}

References [2, 3] highlight the dependence of the measured noise on the total measurement time of a given experiment, t_{meas}. This becomes especially important when comparing noise levels in different devices. For clarity, we report t_{meas} for all appropriate data displayed in this work in Supplementary Table 2.

We note that values of T_2^* used to determine the ratios T_2^e/T_2^* and $T_2^{n_\pi}/T_2^*$ are extracted from separate FID experiments with total measurement times $t_{meas} = 166$ seconds and $t_{meas} = 683$ seconds, respectively. Because these experiments have shorter measurement times than the measurements used to determine T_2^e or $T_2^{n_\pi}$, the determined ratios reported in the main text represent underestimations of the true ratios, i.e., we expect T_2^* to decrease with increasing t_{meas}. However, using Equation 3 in Ref. [3], we estimate that this effect has a relatively small impact on T_2^* for all cases, and thus it does not change the arguments presented in the main text.

Experiment	Figures	t_{meas} (s)
Exchange-FID	2a, 2b (bottom panel)	1508
Exchange-FID	2b (top panel)	140
Exchange-FID	2c, 2d	1514
Three-day Exchange-FID	3a	255600
Exchange-Echo (corresponding FID)	4b, 4c (4b)	2398 ± 12 (166)
Exchange-CPMG (corresponding FID)	4d, 4e (4e)	2035 ± 884 (683)
Exchange-FID	S2a	562
Exchange-FID	S2b	563
ΔB_z-FID	S10	413 ± 3
Exchange-Echo (corresponding FID)	S11 (S11)	2419 ± 9 (89 ± 1)

Supplementary Table 2: Total measurement time t_{meas} of data shown in this work.

SUPPLEMENTARY NOTE 3: ΔB_z AND J_{min}

We measure ΔB_z in our S-T_0 qubit by preparing a singlet, pulsing to large ϵ, waiting a variable length of time, and then pulsing back to $\epsilon = 0$ [4]. In line with previous works [5, 6], we observe that ΔB_z increases linearly with the external field at a rate of approximately 8.38 MHz/T [Supplementary Fig. 10]. Recent work [7, 8] has suggested that such Zeeman gradients may result primarily from differences in the electron g-factors at the different spatial locations of the dots. The actual oscillation frequency of the qubit during this pulse sequence depends on the the amount of residual exchange at large ϵ:

$$f_q = \sqrt{\Delta B_z^2 + J_{min}^2}. \tag{1}$$

All data shown in the main text are acquired at $B_{ext} = 500$ mT, where we measure $f_q = 4.16$ MHz. At this field, we estimate that the true value of $\Delta B_z = 3.85$ MHz based on the average determined Hadamard frequency $f_H = 5.45$ MHz. We then use Supplementary Equation (1) to estimate $J_{min} = 1.58$ MHz.

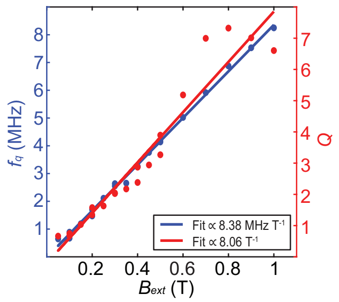

Supplementary Figure 10: **Characterization of the difference in the longitudinal Zeeman splitting at the location of the two dots.** Measurements of $f_q \approx \Delta B_z$ (left axis) and the corresponding oscillation quality factor $Q = f_q \times T_2^*$ (right axis) as a function of B_{ext}. Linear fits to f_q and Q show dependencies of 8.38 MHzT^{-1} and 8.06 T^{-1}, respectively.

SUPPLEMENTARY NOTE 4: CHARGE-NOISE TEMPERATURE DEPENDENCE

We perform spin-echo experiments with J = 20, 35, and 50 MHz as a function of temperature from 50 mK to 325 mK in 25 mK steps. At temperatures above 325 mK, we still observe coherent exchange oscillations, but our measurements suffer from a reduced singlet-initialization fidelity. At each temperature, we recalibrate the H and Z gates that make up the composite X gate used in the spin-echo protocol. We also run an initial FID experiment at each temperature from which we extract values of J and T_2^* as functions of ϵ. We use these extracted values of J to choose the proper values of ϵ such that we evolve at J = 20, 35, and 50 MHz. We analyze the echo measurements following the same procedure used in the base-temperature data analysis.

Supplementary Figure 11 shows the results of the charge-noise temperature dependence. The spectral exponent is relatively insensitive to temperature at each measured J [Supplementary Fig. 11a]. Both T_2^* and T_2^e decrease slightly with temperature, [Supplementary Figs. 11b and 11c]. Moreover, the ratio T_2^e/T_2^* is approximately constant [Supplementary Fig. 11d], which suggests both low- and high-frequency noise have identical temperature dependencies and therefore possibly a common origin.

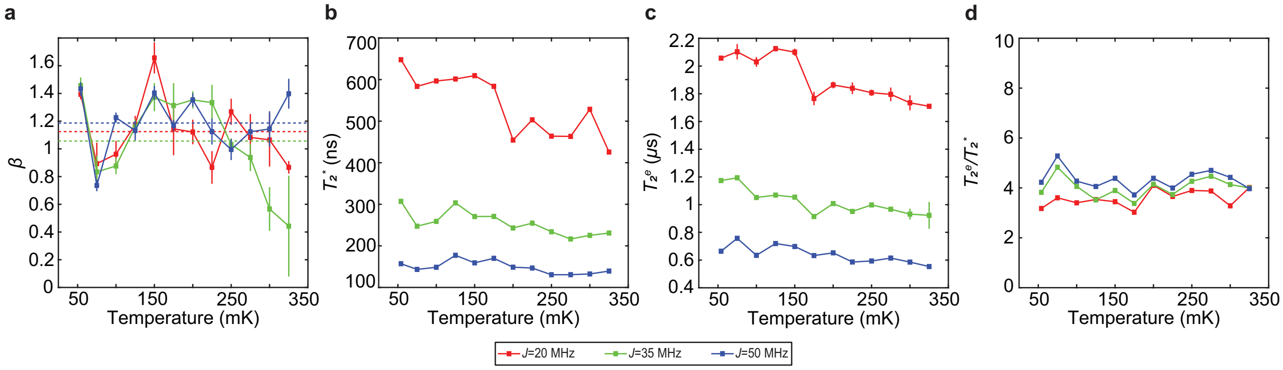

Supplementary Figure 11: **Spin-echo measurements as a function of temperature.** Red, green, and blue data in all plots correspond to data acquired at J = 20, 35, and 50 MHz, respectively. **a** β as a function of temperature. Error bars represent the standard error of the fit for β. **b** T_2^* extracted from corresponding FID experiments as a function of temperature. **c** T_2^e as a function of temperature. Error bars represent the standard error of the fit for T_2^e. **d** Ratio T_2^e/T_2^* as a function of temperature.

SUPPLEMENTARY REFERENCES

[1] O. E. Dial, M. D. Shulman, S. P. Harvey, H. Bluhm, V. Umansky, and A. Yacoby, Charge noise spectroscopy using coherent exchange oscillations in a singlet-triplet qubit, Phys. Rev. Lett. **110**, 146804 (2013).

[2] L. Kranz, S. K. Gorman, B. Thorgrimsson, Y. He, D. Keith, J. G. Keizer, and M. Y. Simmons, Exploiting a single-crystal environment to minimize the charge noise on qubits in silicon, Advanced Materials **32**, 2003361 (2020).

[3] T. Struck, A. Hollmann, F. Schauer, O. Fedorets, A. Schmidbauer, K. Sawano, H. Riemann, N. V. Abrosimov, Ł. Cywiński, D. Bougeard, *et al.*, Low-frequency spin qubit energy splitting noise in highly purified 28 si/sige, npj Quantum Information **6**, 1 (2020).

[4] J. R. Petta, A. C. Johnson, J. M. Taylor, E. A. Laird, A. Yacoby, M. D. Lukin, C. M. Marcus, M. P. Hanson, and A. C. Gossard, Coherent manipulation of coupled electron spins in semiconductor quantum dots, Science **309**, 2180 (2005).

[5] K. Eng, T. D. Ladd, A. Smith, M. G. Borselli, A. A. Kiselev, B. H. Fong, K. S. Holabird, T. M. Hazard, B. Huang, P. W. Deelman, *et al.*, Isotopically enhanced triple-quantum-dot qubit, Science Advances **1**, e1500214 (2015).

[6] J. Kerckhoff, B. Sun, B. Fong, C. Jones, A. Kiselev, D. Barnes, R. Noah, E. Acuna, M. Akmal, S. Ha, *et al.*, Magnetic gradient fluctuations from quadrupolar 73 ge in si/si ge exchange-only qubits, PRX Quantum **2**, 010347 (2021).

[7] R. Ferdous, K. W. Chan, M. Veldhorst, J. Hwang, C. Yang, H. Sahasrabudhe, G. Klimeck, A. Morello, A. S. Dzurak, and R. Rahman, Interface-induced spin-orbit interaction in silicon quantum dots and prospects for scalability, Physical Review B **97**, 241401 (2018).

[8] Y.-Y. Liu, L. Orona, S. F. Neyens, E. MacQuarrie, M. Eriksson, and A. Yacoby, Magnetic-gradient-free two-axis control of a valley spin qubit in si$_x$ge$_{1-x}$, Phys. Rev. Applied **16**, 024029 (2021).
